# Cytotoxicity of the Defensive Secretion from the Medicinal Insect *Blaps rynchopetera*

**DOI:** 10.3390/molecules23010010

**Published:** 2017-12-21

**Authors:** Huai Xiao, Jian-Wei Dong, Di-Jiao Zhou, Xiu-Mei Wu, Jian-Rong Luo, Cheng-Gui Zhang, Na-Na Guo, Yue Li, Le Cai, Zhong-Tao Ding

**Affiliations:** 1 Functional Molecules Analysis and Biotransformation Key Laboratory of Universities in Yunnan Province, School of Chemical Science and Technology, Yunnan University, Kunming 650091, China; Xiaohuai10@263.net (H.X.); jwdongyn@outlook.com (J.-W.D.); 13648847405@163.com (D.-J.Z.); caile@ynu.edu.cn (L.C.); 2 Yunnan Provincial Key Laboratory of Entomological Biopharmaceutical R&D, Dali University, Dali 671000, China; wxm6865@163.com (X.-M.W.); ljrrong@vip.sina.com (J.-R.L.); chenggui_zcg@163.com (C.-G.Z.); guonana000@126.com (N.-N.G.); liyue0208@163.com (Y.L.); 3 National-Local Joint Engineering Research Center of Entomoceutics, Dali 671000, China; 4 Basic Medical College, Chengde Medical University, Chengde 067000, China

**Keywords:** *Blaps rynchopetera* Fairmaire, cytotoxicity, the defensive secretion, benzoquinones, hydroquinone

## Abstract

*Blaps rynchopetera* Fairmaire has long been used as a folk medicine by the Yi and Bai ethnic groups in China to treat fever, cough, gastritis, boils, and tumors. In the present study, the cytotoxicity of the defensive secretion (TDS) of *B. rynchopetera* against AGS Caco-2, HepG2 U251 and Bel-7402 was tested, and the results revealed that TDS had potent cytotoxicity against testing cells with IC_50_ values of 45.8, 17.4, 53.6, 98.4 and 23.4 μg/mL, respectively. Gas chromatography-mass spectrometry (GC-MS) analysis was employed to clarify the cytotoxic constituents in TDS of *B. rynchopetera* and five volatile compounds, including 2-ethyl-2,5-cyclohexadiene-1,4-dione (**3**, 31.00%), 1-tridecene (**5**, 28.02%), 2-methyl-2,5-cyclohexadiene-1,4-dione (**2**, 22.86%), hydroquinone (**4**, 1.33%), and *p*-benzoquinone (**1**, 1.01%), were identified. Chemical constituent investigation on TDS further supported the presence of 5 above compounds. A cytotoxic assay indicated that compounds **1**, **2**, **3** and **4** exhibited significant cytotoxicity against the testing cell lines, implying that benzoquinones and hydroquinone played important roles in the cytotoxicity of TDS of *B. rynchopetera*. TDS is a cytotoxic natural material and further studies investigating mechanisms and inhibitory activities on other cell lines is warranted.

## 1. Introduction

The use of insects as a source of medicines for the treatment of a broad variety of human diseases has a long history in China. The “Compendium of Materia Medica”, the ancient Chinese Pharmacopeia, records more than 100 medicinal insects. Currently, considerable research has investigated insect-derived medicines with the aim of providing scientific evidence for the insects’ proper utilization and modernized development [1,2]. China is one of the earliest countries to exploit insect resources in the world and has been a top producer of many insect-related industrial products for over one thousand years [3]. Insects, such as silkworms and ants, are widely used in the prescriptions of traditional Chinese medicine (TCM). Many pharmaceutical compounds have been identified from the medicinal insects and used for human disease treatments.

*Blaps rynchopetera* Fairmaire belongs to the family Tenebrionidae (Coleoptera) of beetles. This family consists of ca. 10 subfamilies and approximately 20 thousand species with a global distribution. *B. rynchopetera* is traditionally used in the Yunnan Province of China, especially in the areas of Yi and Bai among ethnic minorities [4] for the treatment of fever, cough, gastritis, boils, and even tumors. *B. rynchopetera* is also an edible species with high nutritional value, so in addition to medicinal use, it is used as a snack after roasting. In many farmers’ markets of Yunnan Province, live bugs are sold as farm produce. Our group has studied the chemical components of *B. rynchopetera*, from which we isolated about 20 phenolic compounds, including five new phenolic compounds, rynchopeterines A–E [5] and ten cyclodipeptides [6]. Quantitation of the total polyphenols as the primary anti-oxidant ingredients was performed by comparing two air-dried materials, fresh insect bodies and their defensive secretion liquids [7]. Bioactivity screening revealed that the ethanol extract and the further separated components, petroleum fraction and chloroform fraction of *B. rynchopetera* had strong antineoplastic activity [8]. In recent years, Yan and colleagues also studied another *Blaps* insect, *B. japanensis*, and reported four new compounds, named blapsols A–D, and several other known constituents with inhibitory effects towards COX-1 and COX-2 [9]. Two new products, blapsins A and B, exhibited potent small-molecule 14-3-3 protein–protein interactions (PPIs) [10]. In addition, braeteanolide A, as well as another nine known constituents, showed inhibitory activity against the production of nitric oxide in LPS-induced macrophage cells [11]. 

**Figure 1:**
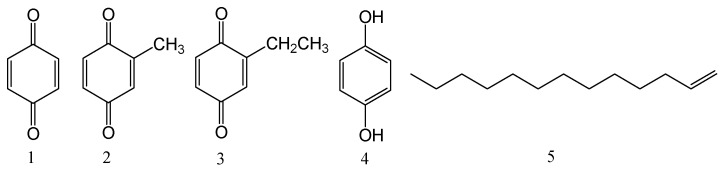
Structures of compounds **1**–**5**.

It is reported that the defensive secretions (TDS) of many insects have rich biological activities [12], including antibiosis [13] and antioxidation [14]. *B. rynchopetera* is known as ‘smelly fart bug’ for releasing a defensive secretion, which is a weapon against predators when stimulated. However, there have been no studies examining antineoplastic activity of TDS of *B. rynchopetera*. The research on TDS of *B. rynchopetera* is pivotal for the development of medicinal drugs. This paper describes cytotoxicity screening of TDS against AGS, Caco-2, HepG2, U251 and Bel-7402 cell lines for the first time, and the chemical constituents that were analyzed by GC-MS to identify five primary compounds, *p*-benzoquinone (**1**, 1.01%), 2-methyl-2,5-cyclohexadiene-1,4-dione (**2**, 22.86%), 2-ethyl-2,5-cyclo-hexadiene-1,4-dione (**3**, 31.00%), hydroquinone (**4**, 1.33%), and 1-tridecene (**5**, 28.02%). The presence of all compounds in TDS was further confirmed by chemical constituent isolation and identification research (Figure 1). Moreover, cytotoxicity of the isolated compounds was tested. The results showed that except for compound **5**, all of the other compounds exhibited significant cytotoxicity against the above cell lines, especially the mixtures **2** and **3**. Compounds **2** and **3** possessed higher contents of TDS and should play an important role for the cytotoxicity of TDS. This paper suggests that TDS is a cytotoxic natural material and that benzoquinones and hydroquinone play important roles in cytotoxicity.

## 2. Results and Discussion

### 2.1. Cytotoxicity of TDS Against Human Cancer Cell Lines

**Figure 2:**
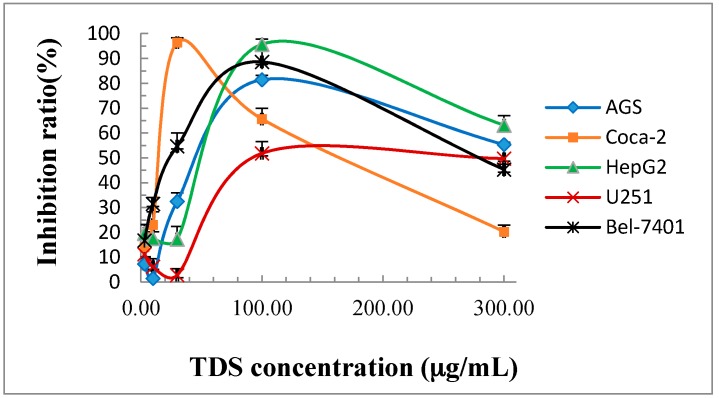
Inhibitory rate curves of TDS on tumor cell lines.

The MTT assays indicated that TDS of *B. rynchopetera* secretion had remarkable cytotoxicity against the growth of AGS, Caco-2, HepG2, U251 and Bel-7402 cells with IC_50_ values of 45.8 ± 5.9, 17.4 ± 2.0, 53.6 ± 5.6, 98.4 ± 4.8 and 23.4 ± 1.2 μg/mL, respectively (Table 1). The highest inhibition rate reached 96.4% on Caco-2 cell line at 30 μg/mL and 95.7% on HepG2 cell line at 100 μg/mL (Figure 2). 

### 2.2. Volatile Components Analysis of TDS

**Figure 3:**
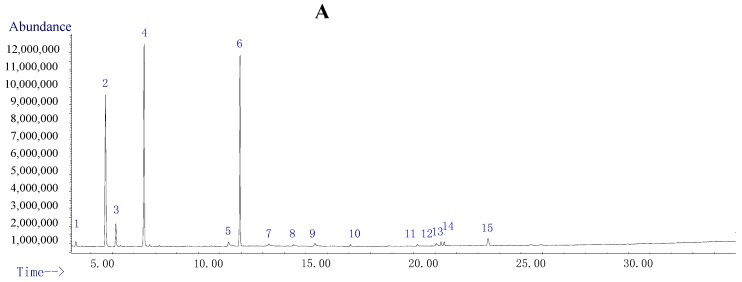

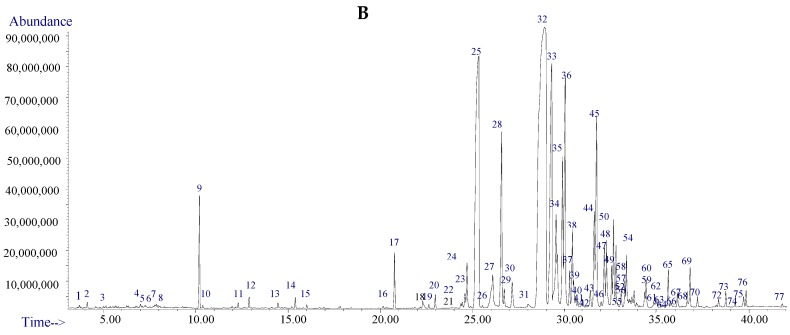
Total ion current chromatogram of TDS (**A**) and the volatile extract of whole body powder (**B**).

The total ion chromatogram (TIC) showed fifteen major peaks in the defensive secretion (Figure 3A), while only five of them were assigned possible structures by NIST (Table 2).

The three compounds with the highest abundance were identified to be 2-ethyl-2,5-cyclohexadiene-1,4-dione (31.00%), 1-tridecene (28.02%) and 2-methyl-2,5-cyclohexadiene-1,4-dione (22.86%), respectively (Table 2). The other two components were identified to be *p*-benzoquinone and hydroquinone. These five components represented 84.22% of the total peak areas. The benzoquinones and 2-methyl- or 2-ethyl-2,5-cyclohexadiene-1,4-dione, two primary components of the defensive secretion of *B. rynchopetera*, were ubiquitous in tenebrionid beetles and other secretory insects [15,16,17,18]. 

### 2.3. Isolation and Structural Identification Using NMR Spectrum

To confirm the above compounds, we further separated and purified TDS with silica gel column chromatography and identified though ^1^D-NMR spectra analysis which confirmed the presence of all five compounds in TDS.

### 2.4. Constituent Comparison between TDS and Volatile Extract from Insect Bodies

To compare TDS constituents with the entire insect body, GC-MS of the volatile extract from insect body powder was performed. The total ion current chromatogram showed seventy-seven peaks in the volatile extract from insect body powder (Figure 3B), of which fifty-six were identified (Table S1). Components with high concentrations (relative amount more than 5%) were identified to be (*E*)-methyl 9-octadecenoate (33.27%), methyl hexadecanoate (15.17%), methyl stearate (8.16%) and ethyl oleate (5.69%), respectively. Fifty-six identified components represented 95.27% of the total peak areas.

Among the five compounds identified in TDS, only 1-tridecene was detected in the extract of the dried body powers. The entire insect body was rich with hydroquinone and its derivatives [7], several of which were isolated and identified by column chromatography [19]. This result showed that the constituents of TDS were highly different from those of the insect body.

### 2.5. Cytotoxicity of Isolated Compounds against Human Cancer Cell Lines

**Figure 4:**
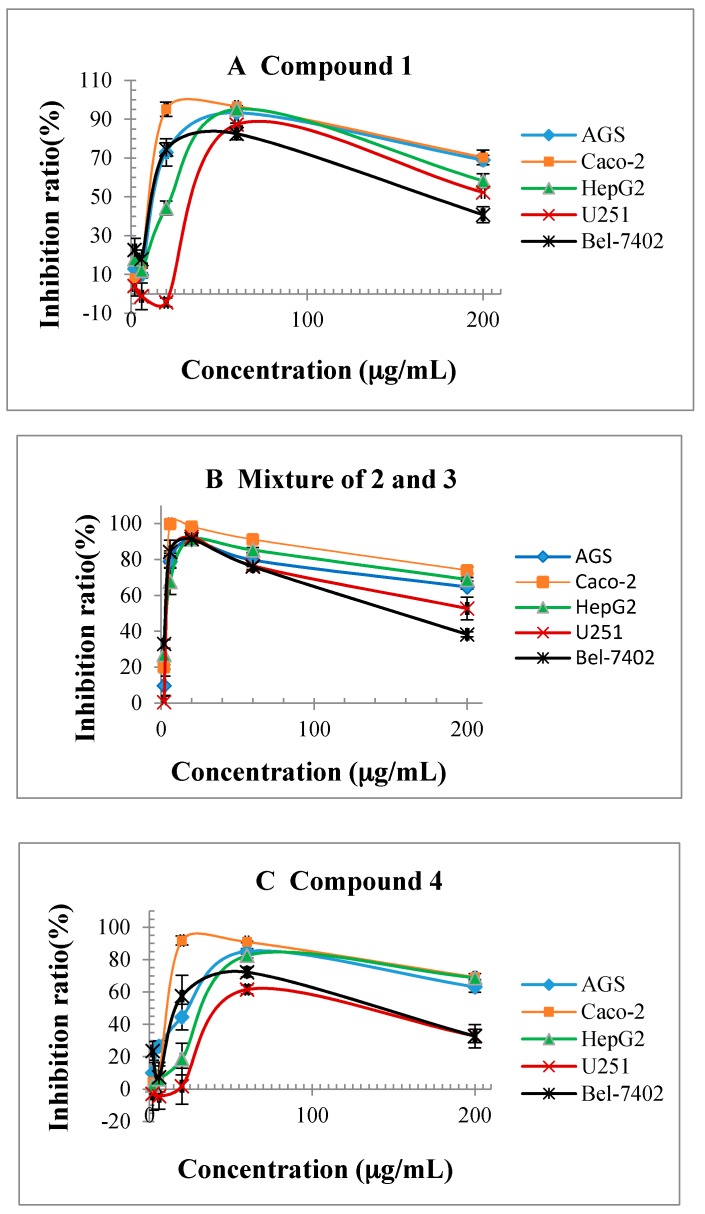
Inhibition of main compounds from TDS for testing cell lines: (**A**) Compound **1**; (**B**) Mixture of **2** and **3**; (**C**) compound **4.**

Previous reports revealed that *p*-benzoquinone and several other derivatives exhibit cytotoxicity [20,21]. The cytotoxicity of the primary constituents of TDS, including a mixture of **2** and **3**, and compounds **1**, **4**, **5** were tested. MTT results showed that the mixture of compounds **2** and **3** had strong growth inhibitory activity for five testing cell lines, AGS, Caco-2, HepG2, U251 and Bel-7402 with IC_50_ 3.6 ± 0.6, 2.8 ± 0.5, 3.8± 0.4, 3.7± 0.8, 2.8 ± 0.2 μg/mL (Table 1), compounds **1** and **4** also had obvious inhibitory effects (Table 1). Compound **5** had no effective influence on testing cell lines. The inhibitory effects of mixtures **2** and **3** were equivalent to the positive control, mitoxantrone with similar IC_50_, one clinical anticancer drug having the same structural characteristics of *para* quinone unit, especially for Caco-2 cell line, inhibition rate reached 99.9% at a concentration of 6 μg/mL (Figure 4).

There was an interesting phenomenon for cytotoxicity testing; all of the testing samples had the same characteristic with an upside—down “U” inhibition ratio curve, inhibitory activity and dosage was positively correlated at low concentrations but changed into negative correlation after an optimum inhibitory concentration.

## 3. Experimental Section

### 3.1. Apparatus

GC-MS was performed with a gas chromatography instrument (Agilent Technologies 7890A, Agilent Technologies, Inc., Wilmington, DE, USA) coupled to a mass spectrometer (Agilent Technologies 5975C, Agilent Technologies, Inc. Wilmington, DE, USA). Compounds were separated on a DB-WAX capillary column (Agilent, 30 m × 0.25 mm, 0.25 µm). NMR spectra were acquired with a Bruker AV-400 spectrometer (Bruker, Karlsruhe, Germany) using TMS as the internal reference.

### 3.2. Materials

Insects were purchased from the farm market of Dali in Yunnan Province, China and were identified to be *B. rynchopetera* Fairmaire by Professor Zi-Zhong Yang at Yunnan Provincial Key Laboratory of Entomological Biopharmaceutical R&D, Dali University. The original specimens (2008071001), were identified by Professor Guo-Dong Ren at the Museum of Hebei University and preserved in Yunnan Provincial Key Laboratory of Entomological Biopharmaceutical R&D, Dali University.

### 3.3. Collection of Defensive Secretion

*B. rynchopetera* live insects were raised in an environment of 18~25 °C with 40~70% humidity. The defensive secretion of the insects was collected during mechanical stimulation. The insects tail part was touched with a small centrifuge tube, and the insect would later secrete defensive liquid directly into the tube. The collected secretion was dissolved with cyclohexane and filtered through a 0.22 µm needle filter to obtain the solution for analysis.

### 3.4. Extraction of the Volatile Extract

Prior to the experiments, insects were sacrificed with ethanol and dried in a drying cabinet at 45 °C and smashed into powder smaller than 50 mesh with a grinder. 5 g dried insect powder was extracted for 30 min with petroleum ether at room temperature, and the process was repeated three times. The extracts were combined and concentrated to obtain the petroleum ether extract. The extract (1 mg) was dissolved with cyclohexane and filtered through a microporous membrane to obtain the volatile extract of the dried insect body.

### 3.5. Cytotoxicity Assays

The defensive secretion and separated fractions were dissolved in DMSO to make 100 mg/mL preparations, which were diluted with phosphate buffer (0.01 M, pH 7.4), reaching a final concentrations of 300, 100, 30, 10 and 3.0 μg/mL, respectively. The positive control samples *cis*-platinum (DDP), mitoxantrone (MA) and the isolated compounds were diluted to final concentrations of 200, 60, 20, 6 and 2 μg/mL.

In vitro antiproliferative activity was assessed by MTT as described [22] with slight modifications. All carcinoma cell lines, including human gastric cancer AGS, colorectal adenocarcinoma Caco-2, liver cancer HepG2, human glioma U251 and human hepatoma Bel 7402 cell lines, were purchased from Shanghai Cell Institute, Chinese Academy of Sciences, Shanghai, China. Bel 7402 cells were seeded in RPMI 1640 medium (pH 7.4) supplemented with 10% (*v*/*v*) fetal bovine serum (FBS), 100 units/mL penicillin and streptomycin in 96-well culture plates at 37 °C in a humidified atmosphere of 5% CO_2_. While AGS, HepG2, and U251 cells were seeded in DMEM medium (pH 7.4) supplemented with the same volume of FBS and antibiotics as above, Caco-2 cells were seeded in DMEM with 15% FBS. Exponential growth cells were trypsinized, counted and diluted to 10,000–30,000 cells/mL.

Cells suspensions (90 μL each) were incubated overnight in a 96-well culture plate and treated with 10 μL sample solutions. After the cells were incubated for 48 h with drugs, the medium was replaced with 15 μL 5 mg/mL MTT and incubated for 4 h followed by adding 150 μL DMSO. The solutions were agitated on a shaker for 5 min and measured for the optical density (OD) at 490 nm wavelength using an ELISA microplate reader. The inhibition (I%) was calculated as follows: I% = (OD_control_ − OD_sample_)/OD_control_ × 100%. The IC_50_ value was calculated by the statistical analysis software SPSS17.0.

### 3.6. GC-MS Analysis

The column temperature was maintained for 1 min at 80 °C during desorption, ramped to 240 °C at 5 °C/min, and kept for 5 min at 240 °C. Splitless injection was conducted, and helium was used as the carrier gas with a flow-rate of 0.9 mL/min. The spectrometer was operated in electron-impact (EI) mode with a scan range of 40 to 400 amu, ionization energy of 70 eV and scan rate of 0.2 s per scan. The transfer line temperature and ionization source temperature were 280 °C and 230 °C, respectively. The volatile components were identified by mass spectral comparison to the spectra of reference compounds in the National Institute of Standards and Technology (NIST) mass spectral library. The approximate relative amounts of individual components were expressed as peak area relative to total peak area.

### 3.7. Isolation and Identification of Compounds in TDS

A total of 4 g of secretion was subjected to silica gel CC eluted with a petroleum/acetone gradient system (50:1 to 1:1) to give six fractions (FrA–FrH). FrA (1.4 g) was further subjected to silica gel CC (petroleum/ethyl acetate: 50:1 to 1:1) to yield compound **5** (609.3 mg). FrB (0.2 g) was further subjected to silica gel CC (petroleum/ethyl acetate: 50:1 to 1:1) to yield compound **1** (9 mg) and a mixture of compounds **2** and **3** (356 mg). FrC (0.4 g) was accomplished by elution with CHCl_3_–CH_3_OH to yield compound **4** (16 mg).

## 4. Conclusions

This paper describes the constituents of TDS of *B. rynchopetera*, which are different from the volatile extracts of insect body powder. Compounds **2** (2-methyl-2,5-cyclohexadiene-1,4-dione) and **3** (2-ethyl-2,5-cyclohexadiene-1,4-dione), which were the primary constituents of TDS, showed strong cytotoxicity against human cancer cell lines, indicating that they play important roles for the cytotoxicity of TDS of *B. rynchopetera*. Further studies investigating mechanisms and inhibitory activities of other cell lines is warranted.

## Figures and Tables

**Table 1:** IC_50_ inhibition of TDS and its main constituents against human cancer cell lines.

Compounds	IC_50_ (μg/mL)/[± Standard Deviation, *n* = 3]				
	AGS	Caco-2	HepG2	U251	Bel-7402
*cis*-platinum (DDP)	2.2 ± 0.3	1.6 ± 0.3	2.0 ±0.2	1.0 ± 0.1	1.7 ± 0.1
mitoxantrone (MA)	2.0 ± 0.2	2.6 ± 0.3	7.7 ± 0.3	0.9 ± 0.1	5.8 ± 0.9
defensive secretion	45.8 ± 5.9	17.4 ± 2.0	53.6 ± 5.6	98.4 ± 4.8	23.4 ± 1.2
**1** (bought sample)	12.8 ± 1.1	10.2 ± 1.3	23.1 ± 4.2	36.5 ± 5.4	11.6 ± 0.9
mixture of **2** and **3** (about 1:3)	3.6 ± 0.6	2.8 ± 0.5	3.8 ± 0.4	3.7 ± 0.8	2.8 ± 0.2
**4**	23.9 ± 0.9	10.8 ± 0.5	33.5 ± 2.1	44.2 ± 3.9	16.1 ± 0.8

**Table 2:** Possible components in the defensive secretion of *B. rynchopetera*.

No.	Retention Time (min)	Content (%)	Compound	Molecular Formula	Molecular Weight	Similarity
1	3.302	1.01	*p*-benzoquinone	C_6_H_4_O_2_	108	95
2	4.684	22.86	2-methyl-2,5-cyclohexadiene-1,4-dione	C_7_H_6_O_2_	122	97
3	5.161	3.51	Not identified			
4	6.484	31.00	2-ethyl-2,5-cyclohexadiene-1,4-dione	C_8_H_8_O_2_	136	93
5	10.394	1.33	Hydroquinone	C_6_H_6_O_2_	110	92
6	10.943	28.02	1-tridecene	C_13_H_26_	182	98
7–15		12.27	Not identified			
Total		100.00				
Total identified		84.22				

## Supplementary Materials

The following are available online, Figure S1: ^1^H-NMR(400M Hz, CDCl_3_) of **2** and **3** in TDS, Figure S2 ^13^C-NMR (100M Hz, CDCl_3_) of **2** and **3** in TDS, , Table S1: Possible components in the volatile extract from insect body powder.
