# Supplementary material for: Cytotoxicity of the Defensive Secretion from the Medicinal Insect Blaps rynchopetera
Source: Molecules. 2017 Dec 21;23(1):10. doi: 10.3390/molecules23010010 (PMC5943921; doi:10.3390/molecules23010010)
Supplement: Supplementary file 1 [file molecules-23-00010-s001.pdf]

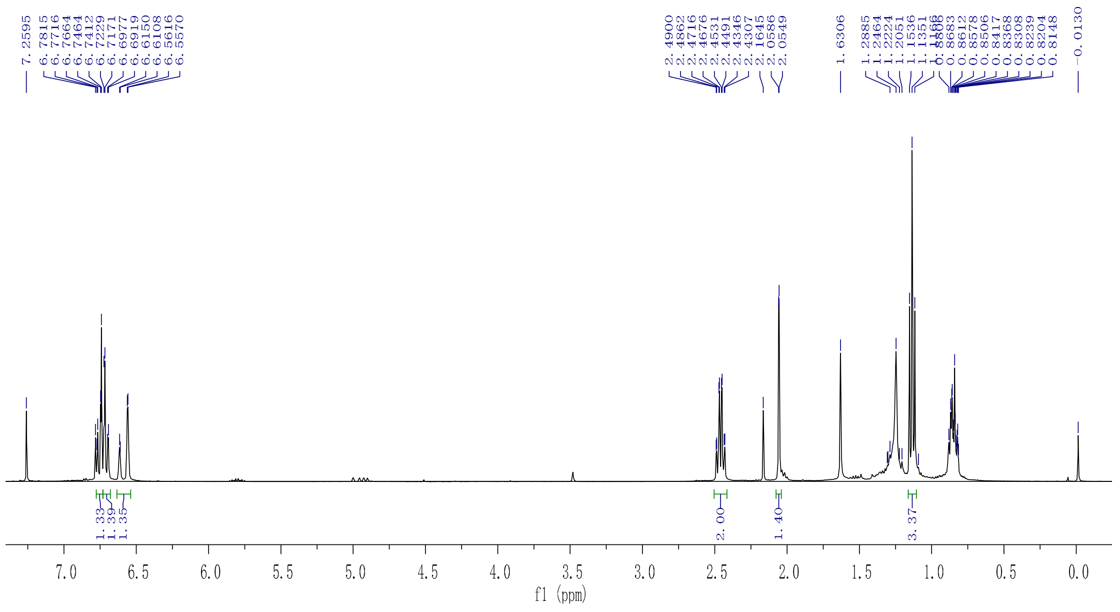

Figure S1: ^{1}H-NMR(400M Hz, $CDCl_3$) of **2** and **3** in TDS.

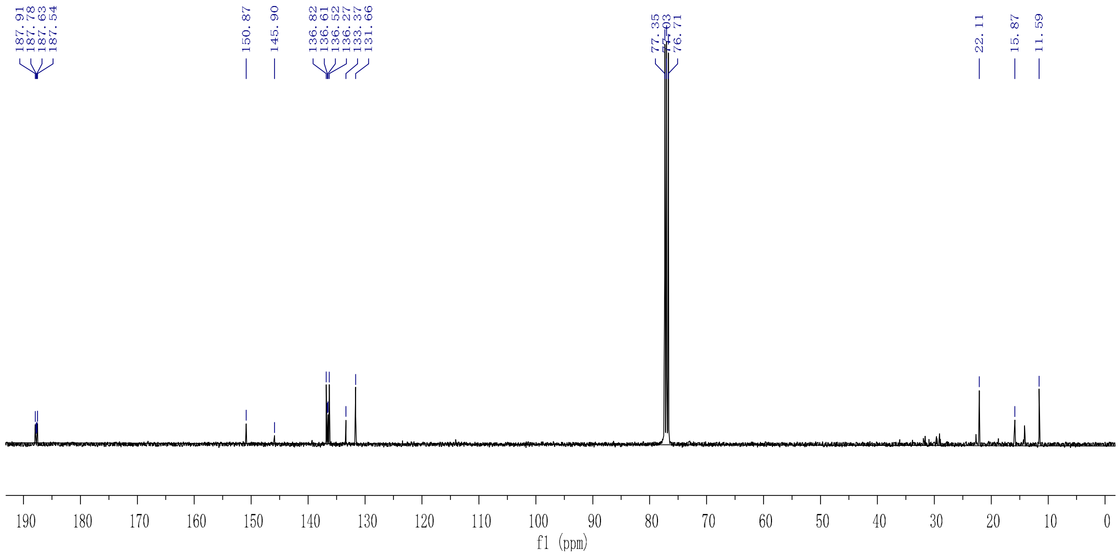

Figure S2: ^{13}C-NMR (100M Hz, $CDCl_3$) of **2** and **3** in TDS.

Table S1. Possible components in the volatile extract from insect body powder.

No.	Retention time (min)	Content (%)	Compound	Molecular formula	Molecular weight	Similarity
1	3.623	0.05	1-ethyl-2-methyl-benzene,	C_9H_{12}	120	95
2	4.052	0.09	mesitylene（1,3,5）	C_9H_{12}	120	95
3	5.066	0.04	1-methyl-3-(1-methylethyl)-benzene,	$C_{10}H_{14}$	134	95
4	6.925	0.07	3-phenylbut-1-ene	$C_{10}H_{12}$	132	90
5	7.199	0.08	not identified			
6	7.700	0.05	naphthalene	$C_{10}H_8$	128	94
7	7.783	0.06	not identified			

8	7.986	0.04	not identified			
9	10.132	1.79	1-tridecene	$C_{13}H_{26}$	182	99
10	10.311	0.05	tridecane	$C_{13}H_{28}$	184	96
11	12.230	0.08	3,7-dimethylnonane,	$C_{12}H_{26}$	170	90
12	12.826	0.16	tetradecane	$C_{14}H_{30}$	198	98
13	14.375	0.10	2,6,10-trimethyl-dodecane	$C_{15}H_{32}$	212	90
14	15.317	0.15	pentadecane	$C_{15}H_{32}$	212	97
15	15.937	0.05	methyl dodecanate	$C_{13}H_{26}O_2$	214	98
16	20.085	0.04	heptadecane	$C_{17}H_{36}$	240	95
17	20.705	0.86	methyl tetradecanoate	$C_{15}H_{30}O_2$	242	99
18	22.219	0.19	ethyl tetradecanoate	$C_{16}H_{32}O_2$	256	96
19	22.553	0.06	not identified			
20	22.910	0.21	methyl pentadecanoate	$C_{16}H_{32}O_2$	256	99
21	24.293	0.09	not identified			
22	24.365	0.11	methyl 7,10-hexadecadienoate	$C_{17}H_{30}O_2$	266	99
23	24.520	0.29	(Z)- methyl 7-hexadecenoate	$C_{17}H_{32}O_2$	268	99
24	24.627	0.88	(Z)- methyl 9-Hexadecenoate	$C_{17}H_{32}O_2$	268	99
25	25.271	15.17	methyl hexadecanoate	$C_{17}H_{34}O_2$	270	98
26	25.461	0.04	not identified			
27	26.022	1.46	hexadecanoic acid	$C_{16}H_{32}O_2$	256	98
28	26.498	3.78	ethyl hexadecanoate	$C_{18}H_{36}O_2$	284	98
29	26.642	0.30	cis- methyl 10-heptadecenoate	$C_{18}H_{34}O_2$	282	99
30	27.071	0.62	methyl heptadecanoate	$C_{18}H_{36}O_2$	284	98
31	27.941	0.14	not identified			
32	28.823	33.27	(E)- methyl 9-octadecenoate	$C_{19}H_{36}O_2$	296	99
33	29.216	8.16	methyl stearate	$C_{19}H_{38}O_2$	298	99
34	29.455	2.13	2-methylpropyl hexadecanoate	$C_{20}H_{40}O_2$	312	95
35	29.800	3.06	ethyl 9,12-octadecadienoate	$C_{20}H_{36}O_2$	308	99
36	29.943	5.69	ethyl oleate	$C_{20}H_{38}O_2$	310	99
37	30.182	0.55	butyl hexadecanoate	$C_{20}H_{40}O_2$	312	99
38	30.337	1.42	ethyl octadecanoate	$C_{20}H_{40}O_2$	312	99
39	30.504	0.13	not identified			
40	30.623	0.10	not identified			
41	30.837	0.07	isopropyl stearate	$C_{21}H_{42}O_2$	326	98
42	30.897	0.12	methyl nonadecanoate	$C_{20}H_{40}O_2$	312	99
43	31.302	0.34	not identified			
44	31.517	2.08	n-propyl 9,12-octadecadienoate	$C_{21}H_{38}O_2$	322	98
45	31.648	3.93	n-propyl 11-octadecenoate	$C_{21}H_{40}O_2$	324	93
46	31.898	0.09	not identified			
47	32.065	1.21	propyl octadecanoate	$C_{21}H_{42}O_2$	326	95
48	32.184	1.17	(E)-6-octadecenoic acid	$C_{18}H_{34}O_2$	282	97
49	32.459	0.62	(Z,Z)-9,12-octadecadienoic acid	$C_{18}H_{32}O_2$	280	94
50	32.566	1.46	not identified			
51	32.697	0.98	methyl eicosanoate	$C_{21}H_{42}O_2$	326	99

52	32.983	0.31	2-methylpropyl octadecanoate	$C_{22}H_{44}O_2$	340	91
53	33.174	0.44	butyl 9,12-octadecadienoate	$C_{22}H_{40}O_2$	336	99
54	33.269	0.76	not identified			
55	33.412	0.05	not identified			
56	33.555	0.22	(E)-9-octadecenoic acid	$C_{18}H_{34}O_2$	282	90
57	33.675	0.26	not identified			
58	33.806	0.10	ethyl 15-methyl-hexadecanoate	$C_{19}H_{38}O_2$	298	90
59	34.247	0.25	not identified			
60	34.330	0.50	not identified			
61	34.735	0.13	not identified			
62	34.867	0.21	iso-propyl 9-octadecenoate	$C_{21}H_{40}O_2$	324	91
63	35.057	0.05	tricosane	$C_{23}H_{48}$	324	94
64	35.415	0.04	not identified			
65	35.701	0.06	not identified			
66	35.534	0.58	pentacosane	$C_{25}H_{52}$	352	96
67	36.023	0.25	methyl docosanoate	$C_{23}H_{46}O_2$	354	99
68	36.559	0.26	9-methyl nonadecane	$C_{20}H_{42}$	282	93
69	36.714	0.67	9-octyl heptadecane	$C_{25}H_{52}$	352	94
70	37.108	0.18	heneicosane	$C_{21}H_{44}$	296	96
71	38.133	0.04	eicosane	$C_{20}H_{42}$	282	96
72	38.252	0.13	methyl 5,13-docosadienoate	$C_{23}H_{42}O_2$	350	93
73	38.645	0.25	tetracosane	$C_{24}H_{50}$	338	98
74	39.134	0.07	methyl heneicosanoate	$C_{22}H_{44}O_2$	340	91
75	39.611	0.20	octacosane	$C_{28}H_{58}$	394	94
76	39.742	0.26	2-methyl tricosane	$C_{24}H_{50}$	338	91
77	41.709	0.05	not identified			
	Total	100.00				
	Total identified	95.27				
